# A Practical Treatise on Diseases of the Skin

**Published:** 1877-02

**Authors:** 


A Practical Treatise on Diseases of the Skin. By Louis A. Duhring, M.D , Professor of Diseases of the Skin in the Hospital of the University of Pennsylvania; Physician to the Dispensary for Skin Diseases, Philadelphia; author of the Atlas of Skin Diseases, etc. J. B. Lippincott & Co., publishers: Philadelphia. 1877.

Dermatological works are too apt to be characterized by a nomenclature based upon anatomical appearances, at the sacrifice of etiological and pathological considerations. Now treatises on this principle may be understood very easily by those made perfect from a long course of study and experience. They, with every appliance for examination convenient, may be able to analyze and diagnose skin affections with comparative ease and certainty. Not so, however, with the general practitioner. If he is taught by books to diagnose from the color, shape, and arrangement of the eruptions, he will be certain to confuse various affections, while he is forced, at last, to resort to an empirical plan of treatment. What, then, is necessary to simplify this subject?

The author of this work claims as one of its merits the pathological basis of its classification. One class—that of the parasitical—he mentions as classed upon its etiological origin. This is a great stride toward the simplification of dermatological treatises. But not only should the classification be founded on this rational and comprehensive basis; the nomenclature should also be cleared of its obscurity, by the same method of etiological and pathological application.

Hardly has the time arrived, however, for this innovation; and, under the circumstances, we freely recommend the book as the best that we have as yet reviewed.

